# Rupture Risk Assessment for Mirror Aneurysms with Different Outcomes in the Same Patient

**DOI:** 10.3389/fneur.2016.00219

**Published:** 2016-12-05

**Authors:** Zhongbin Tian, Yisen Zhang, Linkai Jing, Jian Liu, Ying Zhang, Xinjian Yang

**Affiliations:** ^1^Department of Interventional Neuroradiology, Beijing Neurosurgical Institute, Beijing Tian Tan Hospital, Capital Medical University, Beijing, China; ^2^Medical Center, Tsinghua University, Beijing, China; ^3^Department of Neurosurgery, Medical Center, Beijing Tsinghua Changgung Hospital, Tsinghua University, Beijing, China

**Keywords:** computational fluid dynamics, hemodynamics, intracranial mirror aneurysms, morphologic, rupture

## Abstract

**Background:**

The purpose of this research was to analyze the effect of morphologic and hemodynamic characteristics on mirror aneurysms in which one ruptured and the other did not, within the same patient, and to identify reliable predictors of rupture.

**Methods:**

We performed three-dimensional angiographic imaging in 56 patients with intracranial mirror aneurysms for computational fluid dynamic studies from January 2009 to December 2015. The ruptured aneurysm simulations were conducted with geometry obtained after rupture. The significance of morphologic and hemodynamic parameters with respect to rupture was analyzed. Multivariate logistic regression analysis was applied to significant parameters to identify independent discriminators.

**Results:**

Three morphologic factors (aneurysm size, aspect ratio, and size ratio) and two hemodynamic factors [time-averaged mean wall shear stress (WSS) and low WSS area] were statistically associated with aneurysm rupture (*p* < 0.05). On multivariate logistic regression, a larger size (OR 2.572, *p* = 0.001) and lower WSS (OR 0.609, *p* = 0.045) were independent significant factors for rupture.

**Conclusion:**

Larger aneurysm size and lower WSS were independently associated with the rupture status of aneurysms. These findings need to be confirmed by large multicenter and multi-population studies.

## Introduction

Unruptured intracranial aneurysms (UIAs) are common, occurring in about 3.2% of the adult population worldwide (1). While previous studies have indicated a relatively low annual risk of rupture for UIAs, the high rate of mortality and morbidity associated with aneurysmal subarachnoid hemorrhage remains daunting, perpetuating the controversy surrounding the management of UIAs (1–4).

The lack of consensus on rupture-related factors associated with UIAs is a limitation to making treatment decisions. To identify rupture-related factors, detailed comparisons of the morphologic and hemodynamic characteristics between ruptured and unruptured aneurysms have been performed. These findings are valuable in the decision-making process in relation to whether and when to treat UIAs. However, the results might be affected by patient-related factors, including age, sex, family history, and medical history (1, 2, 4). It is highly valuable to give careful attention to recognize risk factors for aneurysm rupture. Therefore, mirror aneurysms (paired aneurysms on intracranial arteries that occur symmetrically at corresponding bilateral locations) with different rupture statuses might be an ideal within-patient disease model for the analysis of possible factors linked to aneurysm rupture.

Previous studies analyzing rupture-related factors using the mirror aneurysm model had several limitations, such as a small sample size (5, 6), or analysis limited only to morphology (7) or hemodynamics (5). Therefore, in this research, we performed morphologic and hemodynamic analysis of 56 pairs of mirror aneurysms with different rupture statuses. To the best of our knowledge, this is the largest number of aneurysms studied to identify factors related to intracranial aneurysm rupture.

## Materials and Methods

### Patient Selection

**Figure 1:**
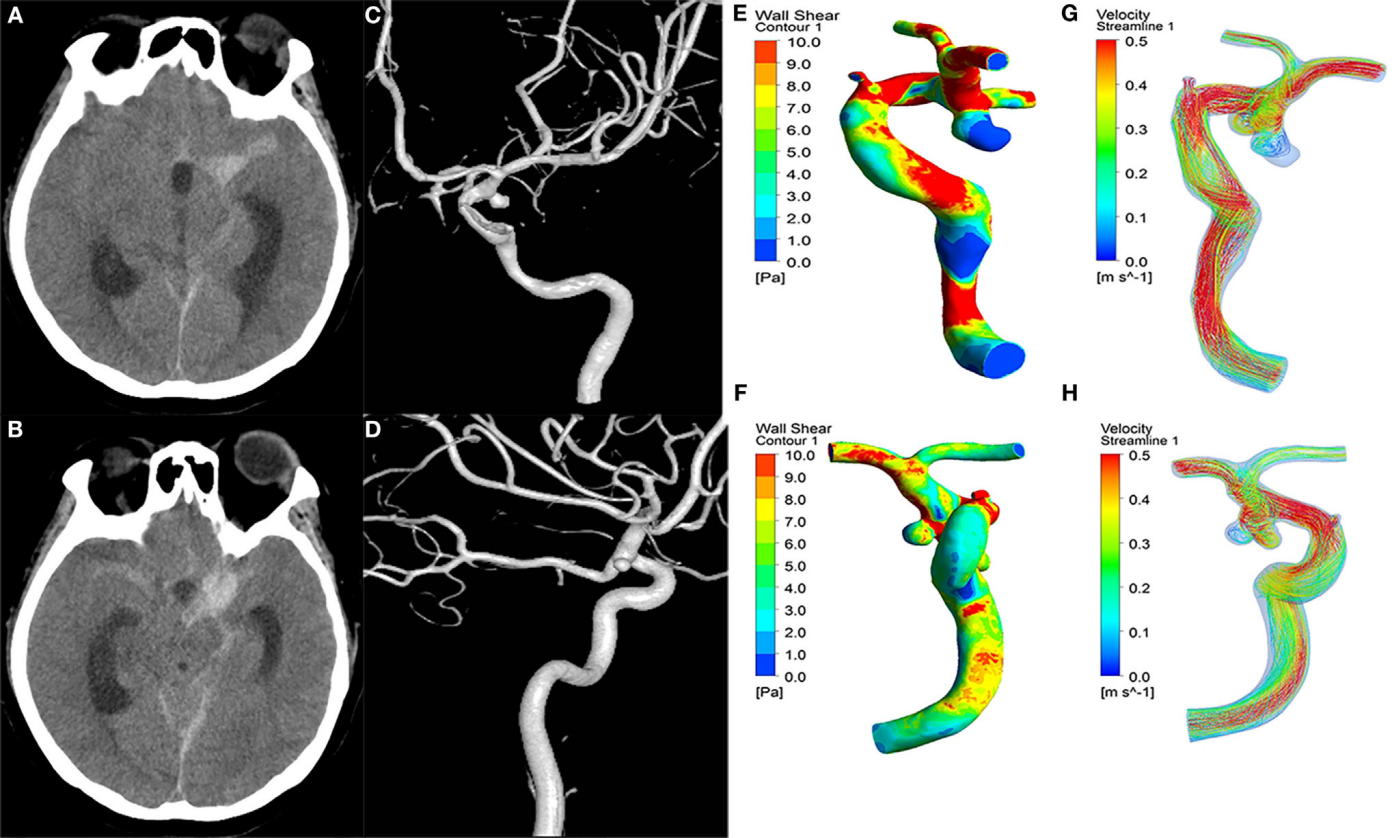
**A 43-year-old female manifested severe headache**. First column **(A,B)**: CT showed subarachnoid hemorrhage. Second column **(C,D)**: the 3D-DSA images presented with two aneurysms on the left ICA C7 (ruptured) and right ICA C7 (unruptured). Third column **(E,F)**: wall shear stress (WSS) distribution. The ruptured aneurysms **(E)** had significantly lower WSS than the unruptured aneurysms **(F)**. Fourth column **(G,H)**: velocity streamlines showing the flow pattern of the aneurysms. The flow patterns of the two aneurysms were simple at peak systole.

Our institutional ethics committee approved this retrospective study. Written informed consents were obtained from patients or their family members during hospitalization. Between January 2009 and December 2015, 152 consecutive patients with intracranial mirror aneurysms (paired aneurysms on intracranial arteries that occur symmetrically at corresponding bilateral locations) were diagnosed in our institute. We selected consecutive patients meeting the following criteria: (1) aneurysms with one ruptured and the other unruptured; (2) rupture identified by intraoperative findings and/or head computed tomography (Figures 1A,B); and (3) 3D digital subtraction angiography (DSA) images of adequate resolution for computed fluid dynamic (CFD) analysis (Figures 1C,D). The following aneurysms were excluded: (1) fusiform or dissecting aneurysms; (2) no history of subarachnoid hemorrhage, or rupture site not able to be identified; and (3) 3D-DSA images too poor for CFD analysis. In total, 56 pairs of mirror aneurysms were included and divided into two groups: ruptured and unruptured. The general information for these patients is summarized in Table 1.

**Table 1:** **Result from univariate statistical analysis for all variables**.

Variables	Total aneurysms (*n* = 112)	Ruptured (*n* = 56)	Unruptured (*n* = 56)	*p-*Value*
Size (mm)	5.00 ± 3.58	6.53 ± 3.50	3.37 ± 2.59	<0.001
Aspect ratio	1.46 ± 0.68	1.84 ± 0.71	1.09 ± 0.37	<0.001
Size ratio	1.87 ± 1.17	2.43 ± 1.27	1.30 ± 0.70	<0.001
WSS (Pa)	3.29 ± 2.30	2.49 ± 1.67	4.10 ± 2.56	<0.001
OSI^a^	0.011 (0.014)	0.013 (0.016)	0.010 (0.011)	0.07
LSA^a^ (%)	4.85 (15.70)	6.78 (27.49)	1.46 (10.20)	0.003
**Location**				0.78
Lateral (%)	53 (47.32)	23 (41.07)	30 (53.57)	
Bifurcation (%)	59 (52.68)	33 (58.93)	26 (46.43)	
**Morphology type**				0.56
Regular (%)	51 (45.54)	21 (37.50)	30 (53.57)	
Irregular (%)	61 (54.46)	35 (62.50)	26 (46.43)	
**Flow stability**				0.14
Stable (%)	47 (41.96)	29 (51.79)	18 (32.14)	
Unstable (%)	65 (58.04)	27 (48.21)	38 (67.86)	
**Flow complexity**				0.08
Simple (%)	68 (60.71)	26 (46.43)	42 (75.00)	
Complex (%)	44 (39.29)	30 (53.57)	14 (25.00)	

*Paired sample t-test, Wilcoxon signed-rank test, or McNemar test as appropriate.

*^a^The data were expressed as median (quartile)*.

### Hemodynamic Models

Computed fluid dynamic numerical simulation of hemodynamics was carried out, as described previously (8, 9). In brief, we performed 3D surface reconstruction using standard proprietary software and saved the surface geometry in standard tessellation language format. The surface data were imported into ICEM CFD software (ANSYS Inc., Canonsburg, PA, USA) to create approximately 3 million finite volume grids and a hybrid was applied with three layers of prismatic grid near walls and tetrahedron grid in the other field to ensure flow was accurately solved. After meshing, CFX V.14.0 software (ANSYS, Inc.) was used for simulation of blood hemodynamics. The governing equations underlying the calculation were the Navier–Stokes formulations, with an assumption of a homogenous, laminar, and incompressible blood flow. A Newtonian fluid condition was used to perform the calculation. The blood vessel wall was assumed to be rigid with no-slip boundary conditions. The average Reynolds number was within the range of normal blood flow in human cerebral arteries, indicating laminar flow condition. The density and dynamic viscosity of blood were specified as ρ = 1060 kg/m^3^ and μ = 0.004 Pa s, respectively. The inflow boundary condition was a pulsatile velocity profile, which was obtained by transcranial Doppler from a healthy subject. The flow waveforms were scaled to achieve a mean inlet wall shear stress (WSS) of 15 dyn/cm under pulsatile conditions (10). Zero pressure was imposed at the outlets. Two cardiac cycles were simulated to allow for numeric stability independent of initialization in the first cycle (11). To confirm numeric stability, the results were collected from the second cardiac cycle only as output for the final analyses. The convergence criteria of simulation are that the residuals are less than 10^−5^ (11, 12).

### Data Collection

The following morphologic parameters were measured and calculated from 3D-DSA data: aneurysm size (maximum height:length from the neck center to the dome of the aneurysm), aspect ratio (dome-to-neck ratio), and size ratio (dome-to-parent artery diameter ratio). The number of sidewall/bifurcation type and regular/irregular type aneurysms were recorded (an aneurysm was defined as being irregularly shaped when blebs, aneurysm wall protrusions, or multiple lobes were present) (13).

Hemodynamic factors were calculated and compared as follows: (1) time-averaged mean WSS; (2) mean oscillatory shear index (14); (3) low WSS area (LSA), defined as the area of aneurysm wall exposed to WSS below 4 dyn/cm (10); and (4) flow pattern: flow stability (a stable flow pattern persisted, whereas an unstable flow pattern moved or changed during the cardiac cycle) and flow complexity (simple flow patterns contained a single vortex, while complex flow had multiple vortices) (15).

### Data Analysis

Statistical analysis was performed using SPSS 17.0 (SPSS Inc., Chicago, IL, USA). Data are expressed as the mean ± SD or median (quartile). The one-sample Kolmogorov–Smirnov test was used to test the normality of the data distribution for quantitative data. Paired sample *t*-tests were used for approximately normally distributed parameters, and Wilcoxon signed-rank test was used for non-normally distributed parameters. For qualitative data, McNemar’s test was performed. *p* <0.05 was considered statistically significant. Then, the factors with *p* ≤ 0.2 in univariable analysis (aneurysm size, aspect ratio, size ratio, WSS, OSI, LSA, flow stability, and flow complexity) were entered into a multivariable logistic regression analysis to assess the independent relationship of all significant univariate factors with aneurysmal ruptures.

## Results

### Clinical Characteristics

The sample comprised 11 males and 45 females who were aged 28–84 (mean 58.89) years. The specific locations of the aneurysms were 12 pairs in the middle cerebral artery and 44 pairs in the internal carotid artery. Thirty ruptured aneurysms were located on the right side and 26 on the left.

### Morphologic and Hemodynamic Characteristics

The ruptured aneurysms were significantly larger in aneurysm size (*p* < 0.001), aspect ratio (*p* < 0.001), and size ratio (*p* < 0.001) than the unruptured aneurysms (Table 1). There were no significant differences between the ruptured and unruptured groups in the lateral/bifurcation or regular/irregular types. For aneurysm hemodynamic factors, the ruptured aneurysms (Figure 1E) showed significantly lower WSS (*p* < 0.001) and higher LSA (*p* < 0.003) than the unruptured aneurysms (Figure 1F). Other hemodynamic factors showed no significant differences between the two groups (Figures 1G,H).

### Multivariate Logistic Regression Analysis

Aneurysm size (OR 2.572; 95% CI: 1.454–4.549; *p* = 0.001) and lower WSS (OR 0.609; 95% CI: 0.372–0.998; *p* = 0.045) were identified as independent predictive factors for mirror aneurysmal rupture (Table 2).

**Table 2:** **Multivariate logistic regression analysis**.

Variables	OR	95% CI	*p*-Value*
Size	2.572	1.454–4.549	0.001
WSS	0.609	0.372–0.998	0.045

*p values for multivariate logistic regression analysis and p < 0.05 was considered statistically significant.

## Discussion

Once a UIA is detected, the decision-making process in relation to whether and when to treat it remains challenging. Clinical, morphological, and hemodynamic features have been considered to identify the association with aneurysm growth and subsequent rupture (16–18). Currently, factors that favor treatment include a young patient with a long life expectancy, previously ruptured aneurysms, a family history of aneurysm rupture, large aneurysms, symptomatic aneurysms, observed aneurysm growth, and established low treatment risks, while conservative management is usually recommended for patients with older age, decreased life expectancy, comorbid medical conditions, and asymptomatic small aneurysms (19, 20). Furthermore, many researchers reported that hemodynamics were thought to play a fundamental role in the formation, progression, and rupture of aneurysms (21, 22). Thus, morphologic metrics and hemodynamic characteristics have been explored in many researches (22–24). These studies suggested that ruptured and unruptured aneurysms have different morphology and hemodynamics. Therefore, analysis of morphologic and hemodynamic parameters between ruptured and unruptured aneurysms is valuable, which may provide an important reference. However, the results might be influenced by patient-related genetics and environmental factors. In an attempt to eliminate bias, we explored the potential rupture mechanisms of mirror aneurysms with different outcomes in the same patient using aneurysmal geometry and hemodynamics analysis. We found that a larger aneurysm size and lower WSS were independent factors associated with mirror aneurysm rupture. These findings were valuable in the decision-making process in relation to whether and when to treat mirror aneurysms and might help physicians to understand the mechanisms of intracranial aneurysms rupture.

In terms of morphology, many parameters (aneurysm size, aspect ratio, and size ratio) were significantly associated with aneurysms rupture by univariate analysis. However, on multivariate logistic regression, aneurysm size was the only independently significant morphological parameter for rupture. Many previous studies concluded that larger aneurysms carry a higher risk of rupture (2, 25). The International Study of UIAs (26) and the Unruptured Cerebral Aneurysm Study of Japan (27) found that intracranial aneurysms of larger than 7 mm were associated with a significant increase risk of rupture. In this study, aneurysms that ruptured were larger than those that unruptured but most were not larger than 7 mm in diameter. This may due to the characteristics of mirror aneurysms themselves, as well as the small sample size. Aneurysm size may be a good assessment factor for mirror aneurysmal rupture, but multicenter studies using larger databases of mirror aneurysms are needed to verify the true situation.

For aneurysm hemodynamic factors, the ruptured aneurysms had significantly lower WSS. The role of hemodynamics in aneurysm rupture is attracting increasing research attention, and a growing number of hemodynamics parameters have been proposed as potential indicators of aneurysm rupture (28). WSS is a frictional force generated by blood flow, which tangentially affects the arterial lumen. It is believed to play an important role in the natural process of cerebral aneurysms. WSS is the most highlighted parameter in recent years (18, 29, 30). It has been reported that ruptured aneurysms have lower WSS magnitude and larger areas with low WSS than unruptured aneurysms (31); it is therefore thought that low WSS may contribute to the process of rupture (32, 33). Accordingly, in the present study, WSS was revealed as an independently significant parameter for rupture by multivariate logistic regression analysis. The ruptured group had lower WSS than the unruptured group, consistent with findings by Lu et al. (5).

Mechanistically, low WSS could trigger mechanobiological processes in endothelial cells, leading to apoptosis and degeneration. Previous studies indicated that WSS was transduced into intracellular signals through mechanical receptors on endothelial cells, subsequently regulating gene expression and blood vessel cell function (10, 32). Low WSS upregulates endothelial surface adhesion molecules, causes dysfunction of flow-induced nitrous oxide, increases the permeability of endothelial cells, and thus, promotes atherogenesis and inflammatory cell infiltration (18, 32). The atherosclerosis and inflammation induced by low WSS may result in degradation of the aneurysm wall that could lead to rupture eventually (34, 35).

The limitations of the present study are its retrospective nature and small sample size. Although 56 aneurysm pairs constitute a small sample with inevitable biases, our findings demonstrate possible characteristics specific to intracranial mirror aneurysms. Moreover, errors in measuring the size of the ruptured aneurysms may be produced and aneurysm geometry may change when the aneurysm rupture. Further studies with larger cohorts from multiple centers are required to verify the morphological and hemodynamic findings in the unruptured aneurysms that eventually rupture. In our CFD simulations, rigid wall, Newtonian fluid, laminar flow, and typical flow waveform of healthy subjects were assumed during the process, which may have an effect on the value of the hemodynamic result. In addition, the mechanisms of mirror aneurysm rupture cannot be clarified simply by morphology and hemodynamics. Further studies are required to analyze more parameters comprehensively.

## Conclusion

Intracranial mirror aneurysms with different rupture statuses within individual patients may be a good disease model with which to assess possible variables related to aneurysm rupture independent of patient characteristics. In the present study, a larger aneurysm size and lower WSS were independently associated with the rupture status of mirror aneurysms. Additional studies are needed to assess to what extent these factors increase the risk of aneurysmal rupture in absolute terms.

## Compliance with Ethical Standards

The ethics committee of our institute approved this study, and informed consent was obtained from each study patient.

## Author Contributions

ZT and YsZ carried out the simulation study and drafted the manuscript. LJ and JL performed data collection and data analysis. XY and YZ participated in the design of this study and helped to check the manuscript.

## Conflict of Interest Statement

The authors declare that the research was conducted in the absence of any commercial or financial relationships that could be construed as a potential conflict of interest.
